# Optochemokine Tandem for Light-Control of Intracellular Ca^2+^

**DOI:** 10.1371/journal.pone.0165344

**Published:** 2016-10-21

**Authors:** Katrin Feldbauer, Jan Schlegel, Juliane Weissbecker, Frank Sauer, Phillip G. Wood, Ernst Bamberg, Ulrich Terpitz

**Affiliations:** 1 Department of Biophysical Chemistry, Max-Planck-Institute of Biophysics, Frankfurt am Main, Germany; 2 Department of Biotechnology and Biophysics, Biocenter, Julius Maximilian University, Würzburg, Germany; 3 Chemical and Pharmaceutical Sciences Department, Johann Wolfgang Goethe University, Frankfurt am Main, Germany; Indiana University School of Medicine, UNITED STATES

## Abstract

An optochemokine tandem was developed to control the release of calcium from endosomes into the cytosol by light and to analyze the internalization kinetics of G-protein coupled receptors (GPCRs) by electrophysiology. A previously constructed rhodopsin tandem was re-engineered to combine the light-gated Ca^2+^-permeable cation channel Channelrhodopsin-2(L132C), CatCh, with the chemokine receptor CXCR4 in a functional tandem protein tCXCR4/CatCh. The GPCR was used as a shuttle protein to displace CatCh from the plasma membrane into intracellular areas. As shown by patch-clamp measurements and confocal laser scanning microscopy, heterologously expressed tCXCR4/CatCh was internalized via the endocytic SDF1/CXCR4 signaling pathway. The kinetics of internalization could be followed electrophysiologically via the amplitude of the CatCh signal. The light-induced release of Ca^2+^ by tandem endosomes into the cytosol via CatCh was visualized using the Ca^2+^-sensitive dyes rhod2 and rhod2-AM showing an increase of intracellular Ca^2+^ in response to light.

## Introduction

In the last decade, optogenetics emerged as a technique that allows the manipulation of cells by light in a broad range of topics such as the restoration of vision after retina degeneration [1], T-cell migration [2], and cell death [3]. Optogenetics has the potential to contribute to or even substitute traditional drug-based therapies in the future [4]. Recently, optogenetic approaches for the generation of Ca^2+^ signals were described, where light-switchable ligands or customized light-gated Ca^2+^ channels were used to generate Ca^2+^ influx through the plasma membrane upon light activation [5–7], but to date, there is no report on intracellularly acting light-gated calcium switches.

Ca^2+^ is a key signal in cell regulation, modulating the activity of a plenitude of sensitive proteins. Ca^2+^ regulation is involved in many fundamental processes such as egg-fertilization, cell cycle, cell-cell contacts, generation of action potentials, motility, hormonal regulation, and programmed cell death via activation of caspases [8]. Prerequisite for accurate signaling is the exact buffering of Ca^2+^ concentrations within the cell. Ca^2+^ ions entering the cell from the environment are either rapidly buffered by Ca^2+^-binding proteins like calmodulin in the sub-second time scale [9] or removed to the extracellular lumen or into intracellular calcium-stores by membrane bound proteins like Ca^2+^-ATPases [8]. The steep Ca^2+^ gradients between the cytosol (100 nM), endosomes (4–40 μM), lysosomes (~500 μM), and the extracellular lumen (~1 mM) allow for the generation of fast, spatially and temporally modulated Ca^2+^ signals [10]. The non-invasive triggering of this universal signal would enable the manipulation of cell behavior. In consequence, detailed insights into the physiology of the cell might be obtained.

The microbial rhodopsin Channelrhodopsin-2 (ChR2) [11] is a light-gated, inwardly rectifying cation channel from the green alga *Chlamydomonas reinhardtii* located in the plasma membrane. In CatCh, a ChR2 mutant, a single amino acid exchange from *Leu* to *Cys* in position 132 leads to 6-fold increased Ca^2+^ permeability in comparison to the wild type [12]. As Ca^2+^ ions are quickly sequestered or exported into the cell environment, the CatCh-mediated signal is expected to arise mainly in the direct neighborhood of the plasma membrane. Distinct light-triggered, intracellular Ca^2+^ signals would require CatCh to be localized in the membrane of organelles that provide higher Ca^2+^ content than the cytosol. In 2011, we described a gene cassette that, once expressed, combines different rhodopsins in one functional protein thereby ensuring stoichiometric expression of both membrane proteins [13]. In order to optically trigger Ca^2+^ signals inside the cell, a tandem combining the light-gated CatCh with a protein providing distinct intracellular trafficking could be used.

Microbial rhodopsins and G-Protein coupled receptors (GPCRs) are members of the same protein super-family. Both exhibit a 7-transmembrane domain structure, making them suitable for the tandem cassette [13]. GPCRs represent the most ubiquitous family of membrane receptors, play a distinct role in cell regulation, and are important drug targets [14]. A subgroup of GPCRs, the chemokine receptors are activated upon external stimuli by their specific agonists, the chemokines. The activation is then followed by the internalization of the membrane-bound receptor protein via clathrin-mediated endocytosis to transfer the signal into the cell. In the early step of vesicle formation, mediated by the adaptor molecules β-arrestin and clathrin [15], the lumen contains the extracellular liquid. After internalization endosomes are acidified to enable ligand displacement, dephosphorylation, and receptor recycling [16], while Ca^2+^ is released from the endosome into the cytosol through TRPML channels, members of the transient receptor potential (TRP) superfamily of ion channels. In early endosomes the Ca^2+^ concentration is already reduced to 3–40 μM in contrast to about 1 mM in the extracellular environment [10].

The Cxc-motive-chemokine receptor 4 (CXCR4) is an important regulatory protein, of high medical relevance, and strongly involved in cell migration of cancers with poor prognosis, metastasis formation, and virulence of the human immunodeficiency virus [17,18]. Once its endogenous ligand, the stromal derived factor 1 (SDF1), binds to CXCR4, the receptor undergoes a conformational change. The signal is then transferred to the cytoplasmic heterotrimeric G_i_ protein, leading to further downstream signaling cascades [19]. Binding of SDF1 to CXCR4 can be impaired by the antagonist AMD3100 (plerixafor) [20]. After internalization, CXCR4 will either be recycled back into the cell membrane or degraded according to the corresponding cellular pathway [15]. Recently, a photoactivatable version of CXCR4 was used to control chemokine receptor signaling and T-cell migration by light [2].

**Fig 1:**
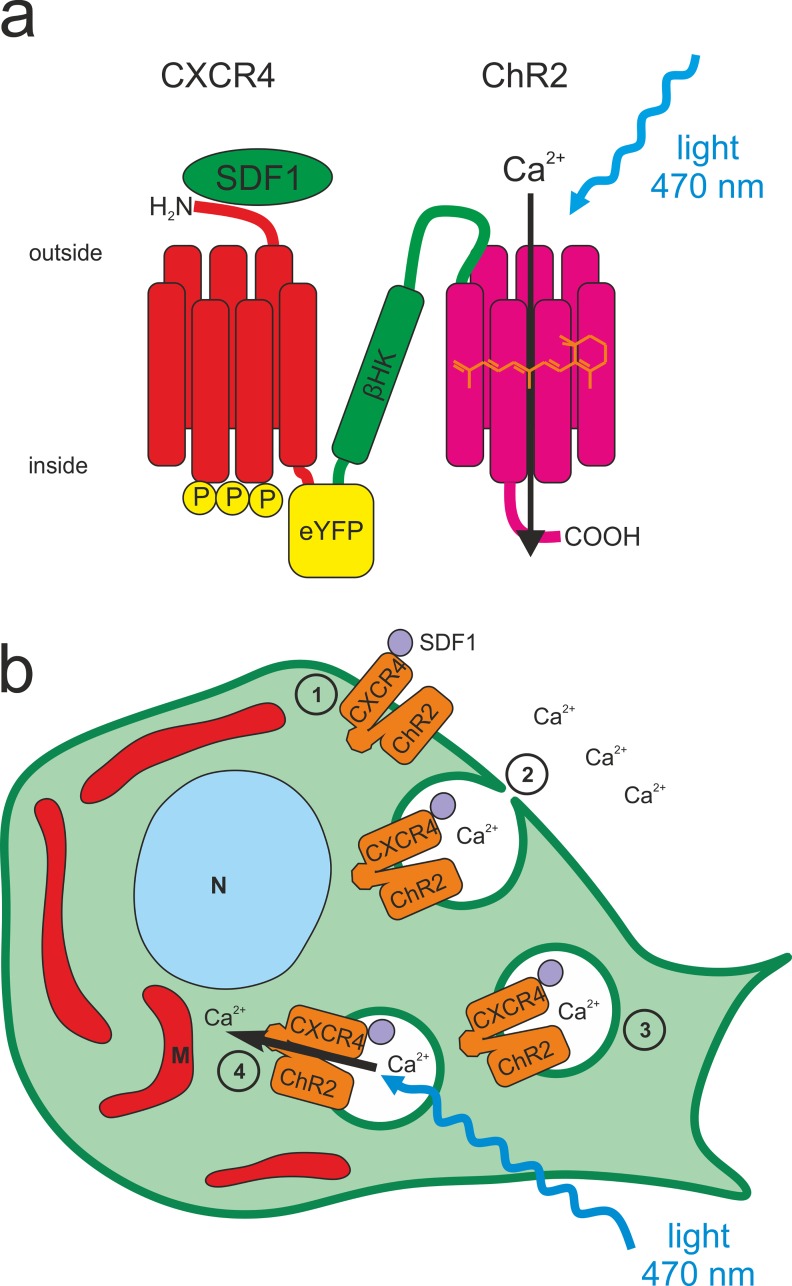
Structure and function of the optochemokine tandem tCXCR4/CatCh. This optophysiological tool combines two features in one functional unit, the internalization after the binding of stromal cell-derived factor 1 (SDF1) and the light-gated influx of cations, including Ca^2+^, via CatCh upon illumination. **a.** The CXCR4-protein was placed at the N-terminus, followed C-terminally by eYFP and the β-subunit of the rat HK-ATPase, to combine the intracellular C-terminus of eYFP with the extracellular N-terminus of ChR2 (aa 1–309). **b.** Schematic overview of the light-induced intracellular Ca^2+^ signaling mediated by tCXCR4/CatCh in an eukaryotic cell. In this optogenetic application the SDF1/CXCR4 signaling pathway (1–4) is used to induce internalization of tCXCR4/CatCh in endosomes. **1.** tCXCR4/CatCh is expressed heterologously in the mammalian cell and trafficked towards and integrated into the plasma membrane. There, the chemokine receptor CXCR4 will be activated by its endogenous ligand SDF1. **2.** Upon SDF1-activation tCXCR4/CatCh is internalized. As the Ca^2+^ concentration in the cell environment is four magnitudes higher than within the cell, also the calcium concentration in these endosomes is considerably higher than in the cytosol. **3.** The endosome is trafficked into intracellular areas of the cell. **4.** Upon illumination with blue light, CatCh will open and release Ca^2+^ ions into the cytosol, resulting in a local intracellular increase of Ca^2+^. This transient Ca^2+^ signal might be used for triggering Ca^2+^-dependent physiological processes by light. N = nucleus, M = mitochondrion.

In the present study we combined CXCR4 with CatCh [12] in one functional tandem protein (Fig 1) which in the following is referred to as the optochemokine tandem tCXCR4/CatCh. tCXCR4/CatCh was overexpressed in the hybrid mouse neuroblastoma × rat glioma cell line NG108-15 and in the human embryonic kidney cell line HEK293. The function of the light-gated cation channel and the internalization of the construct via the endocytotic SDF1/CXCR4 signaling pathway were investigated by patch-clamp measurements and confocal laser scanning microscopy (CLSM). To explore the function of tCXCR4/CatCh the Ca^2+^-sensitive dyes rhod2 or rhod2-AM were used as reporter for changes of the endosomal or intracellular Ca^2+^ concentration, respectively.

## Materials and Methods

### Molecular biology

All constructs and the tandem cassette (Fig 1), which was described in detail before [13], were cloned into pcDNA3.1(-) plasmids (Invitrogen, Carlsbad, USA) under control of the strong human cytomegalovirus (CMV) promoter suitable for mammalian cell transfection. *hcxcr*4 was synthesized (Sloning BioTechnology, Puching, Germany) and introduced into the original tandem cassette (*hvChR1-mKateA-*β*-hChR2*) via *BamH*I and *Sac*II restriction sites to form *hCXCR4-mKateA-*β*-hChR2*. *mKateA* (Evrogen-TagFP635) was then replaced by *eyfp* (*Sbf*I & *Sac*II). Furthermore, a fluorescent variant of hCXCR4 was produced by introducing the construct into a plasmid containing the *eyfp* gene. pcDNA3.1(-)-*hchr2*::*eyfp* and pcDNA3.1(-)-*catch*::*eyfp* were described in an earlier study [12]. DNA sequences of ChR2 and CXCR4 were adapted to human codon usage, to improve recombinant expression in mammalian cells. Plasmids were purified using an endotoxin free purification kit (Nucleobond Xtra Midi EF (Macherey & Nagel, Düren, Germany) or Qiagen Endofree Plasmid Maxi (Qiagen, Hilden, Germany). For establishment of the stable HEK293 Flp-In™T-Rex™ (Invitrogen, Darmstadt, Germany) cell lines, the tandem cassette and *hcxcr4*::*eyfp* were cloned into pcDNA5/FRT/TO using the In-Fusion^®^ HD Cloning Kit (Clontech, Mountainview, USA). DNA constructs are available from the corresponding author upon request.

### Cell culture and Transfection

NG108-15 cells (fused mouse N18TG2 neuroblastoma and rat C6BU1 glioma) were obtained from ATCC. HEK293 Flp-In™T-Rex™ was obtained from Invitrogen. Cells were cultured at 37°C and 5% CO_2_ in Dulbecco's Modified Eagle's Medium (DMEM) supplemented with 4500 mg/l glucose, 10% fetal calf serum (FCS), 2 mM L-glutamine, 100 U/ml penicillin, and 100 μg/ml streptomycin (enriched DMEM). HEK293 Flp-In™T-Rex™ cells were selected using 15 μg/ml blasticidin and 100 μg/ml zeocin before transfection, whereas after transfection zeocin was replaced by 100 μg/ml hygromycin. Sub-confluent cultures (70–90% confluency) were passaged twice a week in a ratio of 1:5 to 1:10 and detached either mechanically or by means of 0.05% trypsin in presence of 0.53 mM EDTA (2 min, RT). G1-Subpopulations were excluded by FACS analysis (S1 Fig). Occurrence of mycoplasms in the cell culture was excluded by frequent PCR-based analysis (LookOut^®^Mycoplasma PCR Detection Kit, Sigma-Aldrich, Germany) of the growth medium and microscopic control.

For recombinant expression of our constructs, NG108-15 cells were seeded either on glass-coverslips (diameter 12 mm) in 24-well plates or in 8-well labtek II (Nunc, Wiesbaden, Germany or Sarstedt, Nümbrecht, Germany) coated with 0.01% Poly-D-lysine and transfected at a cell confluence of 50–80% by means of Lipofectamine® 2000 (Invitrogen, Darmstadt, Germany) in OptiMEM (Invitrogen) medium according to the supplier’s instructions. After a minimum incubation period of 5 h, the medium was exchanged with enriched DMEM including 1 μM *all-trans* retinal (atr). Appropriate protein expression was achieved after 12 to 24 hours.

For generation of stable HEK293 Flp-In™T-Rex™ cell lines of CXCR4::YFP and the tandem construct, cells were transfected using effectene (Qiagen, Hilden, Germany) with the respective pcDNA5/FRT/TO-construct and pOG44 in relation 1:10. The cell line expressing Catch::YFP was available from earlier studies [12].

### Western blot

Membrane proteins (CXCR4::eYFP; tCXCR4/CatCh tandem) were extracted from NG108-15 cells using the Mem-PER Plus Membrane Protein Exctraction Kit (Thermo Scientific, Braunschweig, Germany) 24 to 48h after transient transfection (Lipofectamine® 2000). Before the protein concentration was determined (Quick Start™ Bradford 1x Dye Reagent, BioRad, Munich, Germany; according to manual), membrane protein samples were concentrated (Amicon^®^ Ultracel^®^ 50K, Merck Milipore, Cork, Ireland). Reduced Protein samples (prepared with NuPage LDS Sample Buffer and NuPage Sample Reduction Agent) were separated using Gel electrophoresis (NuPage 10% Bis-Tris Gels) in a XCell SureLock^®^ Mini Cell Chamber (all Invitrogen, Darmstadt, Germany) using Page Ruler Prestained Protein Ladder (Thermo Scientific, Waltham, USA) as a marker. Gels were blotted in an iBlot™ Dry Blotting System (Invitrogen, Darmstadt, Germany) onto a nitrocellulose membrane (Novex iBlot^®^ Gel Transfer Stacks, Invitrogen, Darmstadt, Germany, 23 V, 6 minutes) and blocked overnight at 4°C (dry milk). Primary antibodies and secondary antibodies were incubated for 1 h each: Anti-Mouse CD184(CXCR4) (1:1000, #14-9991-82, eBioscience, Frankfurt, Germany) and Rabbit Anti Rat IgG HRP (1:1000, #PA1-28573, Pierce/Thermo Fisher Scientific). Blots were developed using SuperSignal™ West Pico Substrate (Thermo Scientific, Waltham, USA), Amersham Hyperfilm™ ECL (GE Healthcare, Freiburg, Germany) and a Curix 60 Tabletop Processor (Agfa HealthCare, Bonn, Germany).

### Microscopy

Unless otherwise specified, generally, for fluorescence microscopy 8-well labtek II chambers were used (see cell culture and transfection). Confocal laser scanning microscopy (CLSM) was either performed with a Leica SP2 (Wetzlar, Germany) or a Carl Zeiss LSM 700 (Jena, Germany) microscope. Images were subsequently processed using either LAS AF Lite (v2.6.3., Leica, Wetzlar, Germany) or ZEN 10 (Zeiss, Jena, Germany), and Fiji [21].

The internalization of CXCR4::eYFP and the optochemokine tandem was analyzed after treatment with either 50 nM SDF1α (recombinant human SDF1α; Invitrogen) or 50 nM SDF1α and 10 μM AMD3100 for 40 min *in vivo* or after fixation (10 min; 4% paraformaldehyde) in phosphate buffered saline (PBS, pH 7.4). The anti-mouse CD184 (CXCR4) PE-eFluor^®^ 610 (eBioscience) was added at a ratio of 1:500 (V/V) for 30 min and washed twice with enriched DMEM before measurements. Alexa-647 labelled transferrin was purchased from Thermo Fisher Scientific (Molecular Probes Invitrogen, Darmstadt, Germany) and the staining procedure was performed as recommended by the supplier’s protocol. Early endosomes were visualized using CellLight® Early Endosomes-RFP BacMam 2.0 kit (life technologies—molecular probes) in a concentration of 10 to 50 particles per cell. Cells were imaged after an incubation period of 20 hours.

In order to improve the signal, calcium imaging was performed with tCXCR4/CatCh(D156C) containing the ChR2 L132C-D156C mutant. This mutant exhibits slower kinetics as well as better expression which was beneficial for calcium imaging conditions. tCXCR4/CatCh(D156C) was heterologously expressed in NG108-15 cells. 24 to 48 hours after Lipofectamine® 2000 transfection, the internalization and dye loading were performed under physiological condition (1.8 mM Ca^2+^) in DMEM without serum supplemented with 50 nM SDF1α and 2 μM rhod2-AM or 5 μM rhod2 for 1 hour. In order to remove extracellular Ca^2+^ and to detect only intracellular signals, the cells were washed 2 min with chelator solution (10 mM glucose, 10 mM EGTA, 110 mM NaCl, 10 mM HEPES, pH 7.4). Calcium imaging was then performed in calcium-free solution (130 mM NaCl, 10 mM HEPES, 2 mM MgCl_2_, 30 mM glucose, pH 7.2) supplemented with 10 μM AMD3100. The cells were simultaneously excited with two laser lines (488 nm 10 mW 2%, 555 nm 10 mW 1%) at the confocal microscope (LSM700) at a frequency of 0.5 Hz in order to activate ChR2 and to detect YFP fluorescence and the rhod2 signal at the same time. All parameters (area, light intensity, photomultiplier, pin hole etc.) were kept constant in all measurements. Time series were analyzed with Fiji [21].

For cell death analysis long-term time-lapse imaging experiments were performed. Cells were seeded on coverslips, treated with 50 nM SDF1α for 45 min, and transferred into a custom-built cell chamber (Nikon BioStation IM). Cells were exposed to enriched DMEM at 37°C, 5% CO_2_, and 95% air humidity and illuminated for 20 min by means of a blue LUXEON Rebel light-emitting diode (470 nm, Phillips, San Jose, USA; 5 mW/mm^2^). In a time span of 60 min fluorescence and phase contrast pictures (illumination with red light) were taken every five minutes with a cooled charge-coupled device (CCD) camera. Data was analyzed with the Biostation IM software (Nikon) and GIMP 2.8.10. Cell death ratios after 60 min were estimated by morphological criteria and compared between the different genetic constructs by means of two-tailed student's t-test (p = 0.05).

### Patch clamp

Patch-clamp experiments were performed at two different setups that were described in detail before [22,23], both were equipped with a 473 nm-DPSS laser (Pusch optotech, Baden Baden, Germany) for the activation of Channelrhodopsin-2. For internalization experiments laser light was coupled into a 0.5 mm diameter fiber and measurements were performed at laser intensities in the sample of >10 mW/mm^2^. Experiments were performed with pipette resistances of 1–2 MΩ (cell attached) or 3–5 MΩ (whole cell; GB150F-8P, Scientific-Instruments, Hofheim, Germany) in a temperature controlled patch-chamber [22] allowing for patch clamp experiments at 34°C. The membrane potential was held at -100 mV and cells were illuminated for 100 ms. Once a gigaseal was established, photocurrents were recorded every 2 min for a maximal duration of 30 min. Cell-attached or whole-cell currents were low-pass filtered at 5 kHz and digitized at a sampling rate of 100 kHz. Data recordings were controlled by the Software Clampex 8.2 or 10.3 (Molecular Devices Co.). Data analysis was performed using Clampfit 10.3 (Molecular Devices Co.) and Origin (OriginLab Corporation, Northampton, USA). All data was normalized to the stationary photocurrent at t_0_ (time = 0).

In cell-attached measurements the standard bath solution (140 mM NaCl, 2 mM MgCl_2_, 2 mM CaCl_2_, and 10 mM HEPES pH7.4) was also applied in the pipette but supplemented either with SDF1α, SDF1β or the inhibitor AMD3100. In whole-cell measurements a pipette solution consisting of 110 mM NaCl, 2 mM MgCl_2_, 10 mM EGTA, and 10 mM HEPES pH 7.4 and the standard bath solution were used.

**Fig 2:**
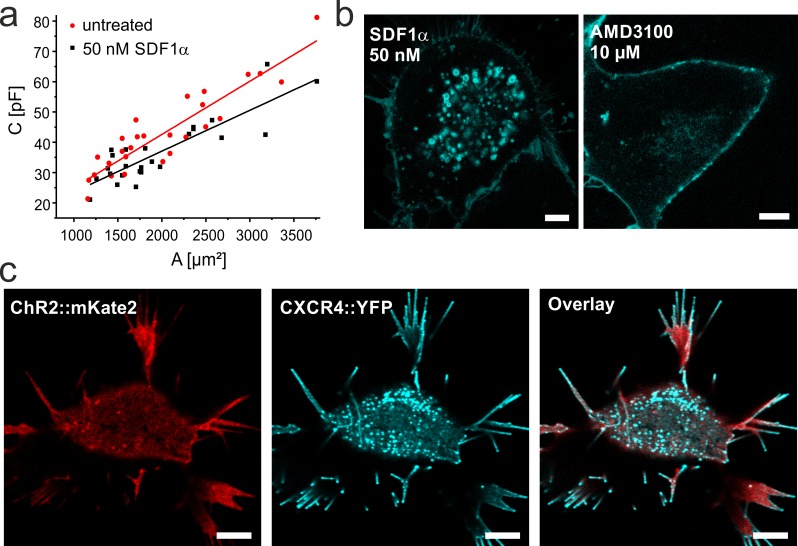
SDF1-mediated CXCR4-internalization in NG108-15 cells. **a.** Plot of cell capacitance (including stray capacitance) measured in whole cell configuration by patch-clamp technique against the membrane area. Cells were either not treated (red) or treated with 50 nM SDF1α (black) for >40 min. The membrane area was calculated from the measured cell diameter assuming a spherical shape of the cell. The specific cell capacitance (slope of the linear fit) decreases in presence of SDF1α. **b.** Confocal laser scanning micrographs of NG108-15 cells expressing CXCR4::eYFP in the presence of the inhibitor AMD3100 or the agonist SDF1α as indicated. While strong internalization is observed with SDF1α, only few vesicles are observed in presence of the inhibitor. **c.** Co-expression of CatCh::mKateA (red) and CXCR4::eYFP (cyan) in presence of 50 nM SDF1α. Note that after binding of SDF1α CXCR4 is internalized while CatCh mainly remains in the plasma membrane and is not directly affected by this growth factor (S3 Fig). Scale bars represent 5 μm.

Cell capacitance measurements in native cells were carried out as described before [24]. Briefly, the capacitive currents during the voltage step were used to define the charges required for the repolarization of the membrane and plotted against the membrane potential. The slope represents the sum of cell and stray capacitance (Fig 2(A)). The membrane surface of each cell was estimated by calculation from the radius assuming a spherical shape. The cell capacitance was plotted against the membrane area and fitted using linear regression. The slope represents the area-specific membrane capacitance.

## Results

### CXCR4::eYFP is internalized in NG-108-15 cells and HEK293 cells

For the functional characterization of tCXCR4/CatCh we chose the NG108-15 cell line, as it is suitable for patch clamp experiments and was successfully used in previous experiments with tandem proteins [13]. NG108-15 cells express endogenous CXCR4 [25] and SDF1-dependent internalization was investigated by capacitance measurements of native cells in whole cell patch-clamp experiments (Fig 2(A)). The membrane capacitance of untreated NG108-15 cells and cells exposed to 50 nM SDF1α was compared. To determine the area-specific membrane capacitance, the respective cell surface was calculated from the radius assuming spherical cell shape. While the area-specific membrane capacitance was 1.72 ± 0.14 μF/cm^2^ (n = 28) for untreated cells, the cell capacitance significantly (α < 0.05, two-tailed student t-test) decreased to 1.35 ± 0.16 μF/cm^2^ (n = 25) after incubation with 50 nM SDF1α for >40 min. The folding factor ϕ [26] describes the folding state of the membrane, reflecting microvilli and other membrane substructures. Thus, ϕ is expected to be higher when no internalization takes place. Indeed, assuming a specific membrane capacitance of 0.8 μF/ cm^2^ ϕ values of 2.15 for untreated and 1.69 for treated cells were calculated. A similar, significant reduction of membrane capacitance was also observed in electrorotation experiments (S2 Fig) confirming our results.

CXCR4 is translocated via the clathrin-mediated endocytic pathway [17]. To visualize the SDF1α-induced internalization process, the NG108-15 cells were transiently transfected with CXCR4 tagged with yellow fluorescent protein (CXCR4::eYFP) and investigated by CLSM. Intense fluorescence in the membrane clearly indicated the successful expression of CXCR4::eYFP in NG108-15 cells (Fig 2(B) and 2(C)). After treatment with 50 nM SDF1α for 40 min, CXCR4::eYFP was partly internalized into small vesicles with diameters of 300–1500 nm (Fig 2(B)). In contrast, when cells were treated with 10 μM of the antagonist AMD3100, the internalization was strongly reduced, indicating a functional CXCR4 internalization system in NG108-15 cells. Similar results could be obtained using HEK293 cells overexpressing CXCR4::eYFP (see Material and Methods; S4 Fig) as an alternative cell system.

The high activity of SDF1α-mediated internalization might lead to unspecific displacement of other membrane proteins. If the specificity of CXCR4 cargo selection in clathrin-coated pits could be impaired by overexpression of the membrane-bound light-gated cation channel Channelrhodopsin-2 (ChR2) it would eventually result in translocation of both proteins into endosomes and thus provide a strategy to localize ChR2 in intracellular vesicles. To test this hypothesis, fluorescently tagged ChR2 (CatCh::mKateA) was co-expressed together with CXCR4::eYFP in NG108-15 cells. As can be seen in the CLSM images, CatCh::mKateA is not substantially internalized upon addition of SDF1α (Fig 2(C); see also S3 Fig) but mainly remains in the membrane, while internalization of CXCR4::eYFP is not affected by the presence of CatCh.

### The optochemokine tandem tCXCR4/CatCh is internalized in presence of SDF1α via clathrin-mediated endocytosis

**Fig 3:**
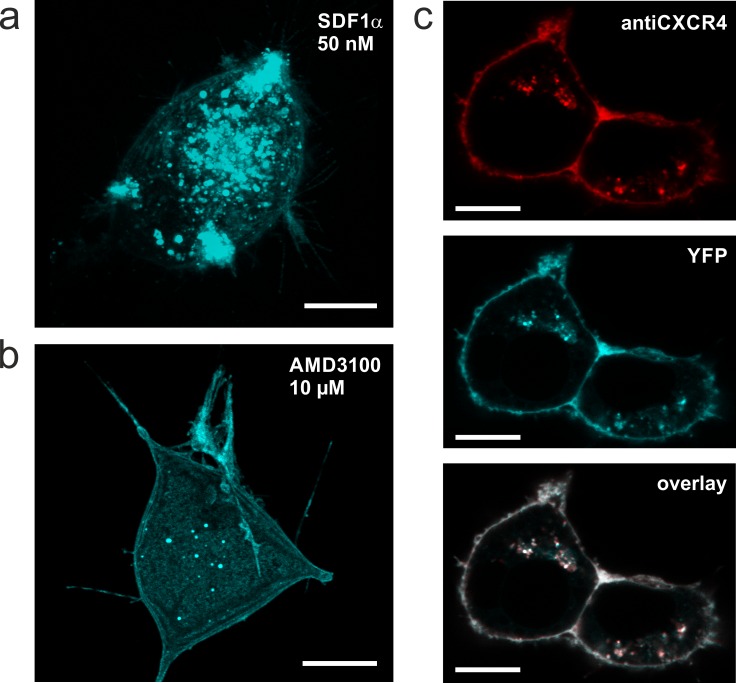
Confocal laser scanning micrographs of tCXCR4/CatCh protein in NG108-15 cells. **a,b.** Z-stack overlay of a typical tCXCR4/CatCh cell either treated with 50 nM SDF1α for 45 min (a) or with AMD3100 (b). The tandem construct is internalized by the action of SDF1α (similar to CXCR4 shown in Fig 2). **c.** Internalization of anti-CXCR4 antibody after immunoreaction with tCXCR4/CatCh in cells exposed to 50 nM SDF1α. The fluorescence signal of anti-CXCR4 (top, red) and tCXCR4/CatCh (middle, cyan) co-localize to a great extend as can be seen from white areas in the overlay (bottom). Scale bars represent 10 μm.

In order to realize mutual internalization of CatCh and CXCR4, both proteins were combined in one functional tandem entity. The optochemokine tandem tCXCR4/CatCh (Fig 1(A)) was either expressed in NG108-15 (Fig 3) or HEK293 cells (S5 Fig). Similarly to earlier investigations of tandem proteins [13], the protein was expressed in one entity and not fragmented during the expression/trafficking process. This was shown by Western blot analysis of membrane proteins isolated from NG108-15 cells expressing tCXCR4/CatCh stained with anti-CXCR4 antibodies (S6 Fig). In agreement with the Western blot results we observed robust expression and localization of the YFP fluorescence in the cytoplasmic membrane for both mammalian cell types.

For our strategy it is fundamental that both 7TM-proteins preserve their function even when combined in one tandem protein. Thus, the functional tandem proteins should be internalized while CatCh should have unaltered kinetics and channel characteristics. The ability of the tandem to be internalized upon addition of SDF1α was shown in transiently transfected NG108-15 cells treated either with 50 nM SDF1 only, or with 10 μM AMD3100. CLSM images clearly showed that after 40 min in presence of SDF1α the majority of tCXCR4/CatCh proteins was located in intracellular vesicles (Fig 3(A)), putative endosomes. The number of vesicles was strongly reduced when cells were treated with the antagonist AMD3100 (Fig 3(B)). Thus, in tCXCR4/CatCh the internalization feature of CXCR4 was not impaired by the additional C-terminally fused CatCh. In average the tCXCR4/CatCh vesicles were bigger (up to 4–5 μm) than CXCR4::eYFP vesicles (<2 μm).

To support our assumption that the vesicles represent endosomes internalized from the membrane, we used a functional CXCR4 specific antibody labelled with red-fluorescent dye (Anti-Mouse CD184 PE-eFluor 610; eBioscience). Immediately after the treatment, immuno-labeled CXCR4 was only found in the cytoplasmic membrane. After several hours of incubation it was also localized in a number of intracellularly located vesicles. These vesicles exhibited both red and green fluorescence indicating successful translocation of the tandem protein from the plasma membrane (Fig 3(C)). Strikingly, the antibody did not show any immunoreaction with endogenous NG108-15 and HEK293 CXCR4 neither in microscopic analyses nor in Western blot experiments (S6 Fig) thus allowing for specific detection of the heterologous CXCR4 constructs. The *in vivo* immunostaining of heterologous CXCR4 showed that the majority of the intracellular, green-fluorescent vesicles originated from the cytoplasmic membrane (Fig 3(C)), further supporting the assumption that these intracellular vesicles represent endosomes.

To verify this, NG108-15 tCXCR4/CatCh cells were transfected with fluorescent early-endosome marker Rab5a-RFP [27,28]. Indeed, vesicles that exhibited green and red fluorescence could be observed indicating the internalization of tCXCR4/CatCh into endosomes (data not shown). However, the overall transduction efficiency of the baculovirus Rab5a-RFP construct in NG108-15 cells was very low. Therefore, a similar experiment was performed in stable HEK293 FlpIn-T-Rex^TM^ (Invitrogen) cell lines expressing either CXCR4::eYFP or the tCXCR4/CatCh tandem. Rab5a-RFP colocalized with either CXCR4::eYFP or the tCXCR4/CatCh tandem (S7 Fig), confirming the results in NG108-15 cells. Also alexa647-labeled transferrin, a compound known to be internalized in endosomes [29], was observed to cluster in the same regions as the tandem constructs or Rab5a, hence giving more evidence that the optochemokine tandem is internalized via clathrin-mediated endocytosis into early endosomes (S7 Fig).

### CatCh is functional in tCXCR4/CatCh

**Fig 4:**
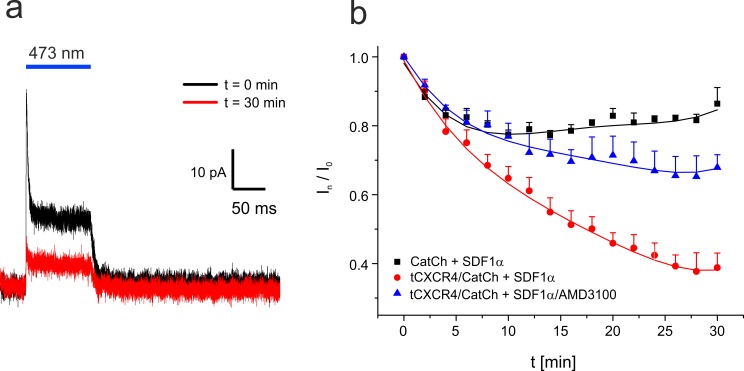
Patch-clamp investigation of the optochemokine tandem in cell attached configuration at 34°C. The pipette solution was supplemented with 50 nM SDF1α. **a.** Typical current trace recorded at an applied membrane potential of -100 mV. Light-dependent signal of tCXCR4/CatCh directly after the sealing process and 30 min later. During that time the cell was illuminated every two minutes for 100 ms. **b.** Time course of the relative tCXCR4/CatCh current in presence of SDF1α (square, 5 cells) or SDF1α and the inhibitor AMD3100 (triangle, 3 cells), and CatCh in presence of SDF1α (circle, 4 cells). Mean values are given, bars represent the standard error.

Once tCXCR4/CatCh is internalized into the cell, it is designed to release cations upon illumination. In order to assess whether CatCh is fully functional in tCXCR4/CatCh, patch-clamp experiments using NG108-15 cells expressing either tCXCR4/CatCh or CatCh::eYFP were performed. After illumination (diode pumped solid-state laser; 473 nm; saturating conditions: >10 mW/mm^2^) we observed the typical CatCh signal [12] in both proteins (Fig 4(A)): A short transient peak, which was followed by a stationary current that decayed with a time constant of 16 ± 3 ms (S8 Fig) indicating unchanged kinetics of CatCh in the tandem protein. The mean current density at -100 mV measured in whole cell conditions in NG108-15 cells was -67 pA/pF (tCXCR4/CatCh; n = 11, s.e.m = 6) and -125 pA/pF (CatCh; n = 14, s.e.m. = 8). Thus, we conclude that CatCh is functionally expressed in the optochemokine tandem and that the expression level of the tandem construct is slightly reduced in comparison to CatCh only.

The functional expression of CatCh allowed us to electrophysiologically monitor the internalization process of tCXCR4/CatCh. In order to avoid possible negative effects of cytosol replacement by pipette solution on the internalization process, the cell attached patch-clamp mode was used ensuring the complete preservation of the cytosol. In these experiments the pipette solution was identical with the extracellular solution supplemented either with 50 nM SDF1α only or in addition to 10 μM AMD3100. The light-triggered channel response was measured every 2 min within a time course of 30 min at about 34°C (Fig 4(B)).

In the presence of SDF1α the signal recorded from tCXCR4/CatCh cells decreased by about 60% within half an hour. In contrast, when the action of SDF1α was impaired by its antagonist AMD3100 the signal decreased only by about 30%. In accordance, cells expressing CatCh::eYFP showed only an initial signal reduction by 15–20% and not further reduction. Thus, also the electrophysiological data clearly indicate functional SDF1 dependent internalization of the optochemokine tandem. We investigated the role of SDF1 splicing variant [30] and the agonist concentration on the internalization behavior of tCXCR4/CatCh. However, no significant differences in internalization kinetics between SDF1α and SDF1β or 50 nM and 100 nM SDF1α, respectively, could be observed (S9 Fig).

### Optochemokine tandem endosomes release calcium into the cytosol upon illumination

Upon SDF1α incubation and illumination of tCXCR4/CatCh expressing cells we expect intracellular Ca^2+^ signals to be generated by ion efflux from endosomes. In order to gain more information on the Ca^2+^ transfer inside the cells, we performed Ca^2+^ imaging experiments. In these experiments we used a variant of tCXCR4/CatCh, in the following referred to as tCXCR4/CatCh(D156C), in which *Asp*156 of CatCh was mutated to *Cys*. This mutation alters the gating behavior of the channel [31] and was recently used in optogenetic experiments with *Drosophila*, renamed as ChR2-XXL due to its prolonged open state and improved expression [32]. These advantages were also observed in NG108-15 cells and allowed us to trigger tCXCR4/CatCh(D156C) with very short light pulses with a scanning laser during CLSM measurements.

**Fig 5:**
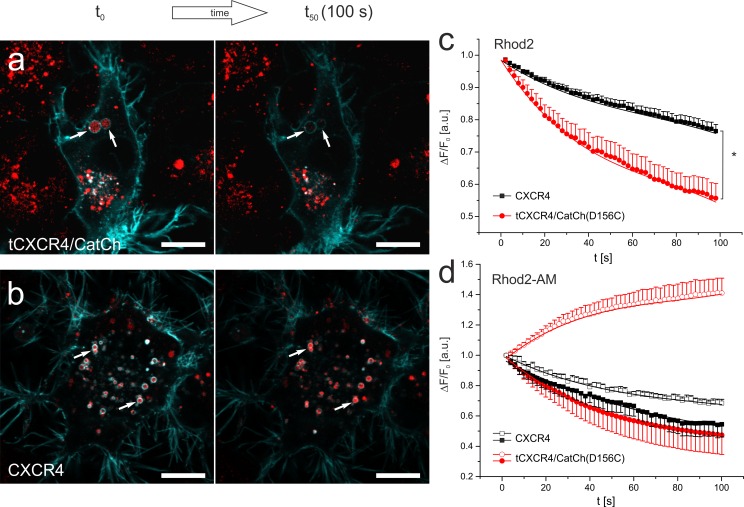
Intracellular, light-induced Ca^2+^-signaling mediated by optochemokine tandem-endosomes. **a,b.** CLSM micrographs of NG108-15 expressing the tCXCR4/CatCh(D156C) (a, cyan) or CXCR4::eYFP (b, cyan) stained with rhod2 (red). White arrows highlight such endosomes that express the respective membrane protein and are loaded with rhod2. Note that the rhod2 intensity decreases in tCXCR4/CatCh(D156C) to a larger extent than in CXCR4::eYFP endosomes. **c.** Time course of the rhod2 fluorescence of endosomes exhibiting tCXCR4/CatCh(D156C) (17 cells, mean s.e.m) or CXCR4 (21 cells) as identified by their fluorescence. Upon blue light activation rhod2-fluorescence non-linearly decreases in endosomes with tandem construct, while endosomes containing CXCR4 in their membrane show only moderate, nearly linear decrease of rhod2 fluorescence. **d.** Time course of rhod2 fluorescence in endosomes (filled symbols) and nuclei (empty symbols) as a measure for small changes in the cytosolic Ca^2+^ [33] of cells loaded with the membrane permeable rhod2AM-derivate (See also S11 Fig). Note that in tCXCR4/CatCh(D156C) cells (red, n = 7, mean + s.e.m) an increase in cytosolic Ca^2+^ concentration was observed while only fluorescence decrease was observed in CXCR4-expressing cells (black, n = 8, mean + s.e.m.). All rhod2-experiments were performed in absence of extracellular Ca^2+^. Scale bars represent 10 μm.

NG108-15 cells either expressing CXCR4::eYFP or tCXCR4/CatCh(D156C) tandem were exposed to 5 μM rhod2 (not membrane permeable) and 50 nM SDF1α in the presence of physiological extracellular Ca^2+^ and subsequently analyzed using CLSM in absence of extracellular Ca^2+^ (Fig 5(A) and 5(B)). Within 60 s after illumination (488 nm laser) the red fluorescence of the tCXCR4/CatCh(D156C) endosomes decreased much faster and in a non-linear fashion compared to endosomes expressing CXCR4 only (Fig 5(C); significant difference after 8s). This observation indicates an accelerated reduction of the endosomal Ca^2+^ concentration mediated by the action of CatCh in the tandem construct. In accordance with this assumption we did not observe any difference when CatCh was illuminated with 640 nm laser (no excitation of CatCh; S10 Fig). In order to discover whether a release of Ca^2+^ from the endosomes could notably change the Ca^2+^ concentration in the cytosol, we performed imaging experiments with cells loaded with 2 μM rhod2-AM. In each cell the fluorescence intensity within one representative endosome and the nucleus (as a measure for small changes in the cytosolic Ca^2+^ concentration [33]) were compared over time (Fig 5(D); S11 Fig). Indeed, in tCXCR4/CatCh(D156C) endosomes we observed a decrease of fluorescence while the fluorescence in the nucleus increased. In contrast, the CXCR4 cells exhibited a decrease of fluorescence in nucleus as well as endosomes.

## Discussion

To date there is no report about functional expression of intracellularly acting, light-controlled Ca^2+^ regulators. This is due to the absence of suitable strategies for the intracellular expression of light-switchable membrane-transport proteins. The light-induced release of Ca^2+^ from intracellular calcium stores would enable the precise regulation of physiological processes and thus are expected to be of extreme interest for cell biologists.

Herein, we established a novel approach for the light-controlled, intracellular manipulation of the second messenger Ca^2+^ by re-engineering our previous invention, a gene cassette for stoichiometric expression of two rhodopsins [13]. The novel optochemokine tandem was designed to shuttle the tool from the plasma membrane into intracellular organelles by ligand-induced endocytosis, to monitor the displacement by decreasing ion-channel activity, and to generate intracellular Ca^2+^ elevation by light. N-terminally the chemokine receptor CXCR4 was set to take advantage of the well-known clathrin-mediated endocytosis [17,30] and to traffic the optochemokine tandem into intracellular organelles in a SDF1-dependent manner. As optogenetic actuator the CatCh mutant of the light-gated cation channel ChR2 was placed c-terminally. This mutant holds an about 6-fold enhanced Ca^2+^ permeability [12] compared to the wild type protein. In addition, the light-sensitivity of this actuator was further improved by the point mutation D156C [31,32], which exhibits extra high expression and a long open state.

Two mammalian cell lines were selected for the characterization of this novel optophysiological tool after testing for the SDF1-dependent internalization of CXCR4 by capacitance measurements and fluorescence microscopy (Fig 2 and S4 Fig). Our data clearly shows that tCXCR4/CatCh was functionally expressed in NG108-15 and HEK293 cells, that the activation of CXCR4 by SDF1 in tCXCR4/CatCh led to the internalization of the whole tandem construct by endocytosis via the clathrin-mediated endosome pathway, and that the optophysiological tool could generate intracellular Ca^2+^-elevation upon light-induced channel opening. In contrast, co-expressed single fluorescent CXCR4 and CatCh constructs did not lead to any substantial internalization of the cation channel.

Similar to other rhodopsin-tandem proteins [13] tCXCR4/CatCh was expressed as one single fluorescent entity (S6 Fig). From the whole-cell patch-clamp data we conclude that the opotchemokine tandem in contrast to other previous tandems was expressed to a similar extent as CatCh::eYFP. tCXCR4/CatCh located both in the plasma membrane and in intracellular vesicles. By immune-labeling with a red-fluorescent CXCR4-specific antibody we identified the majority of intracellular vesicles as tCXCR4/CatCh endosomes, which had been internalized from the plasma membrane within several hours after staining of the plasma membrane (Fig 3(C)). Thus, internalization behavior of tCXCR4/CatCh was similar as in the pure CXCR4::eYFP constructs (Fig 2(B)). The internalization process could, to a great extent, be inhibited by the specific antagonist AMD3100 which blocks SDF1-binding [20]. In addition, our experiments with the endosome markers Rab5 and transferrin clearly showed that the internalization process is realized via the well-conserved clathrin-pathway [34] (S7 Fig).

Also the kinetics of the internalization process observed in patch clamp experiments analyzing the photocurrent over time was in accordance with previously published data measured in other cell types [35,36]. Interestingly, with this sensitive method we could not detect any significant difference between the splicing variant alpha or beta of the SDF1 ligand [30] (S9 Fig), suggesting successful activation of the tool by both variants. SDF1-specificity of endocytosis was further supported by the fact that in presence of the antagonist AMD3100 the internalization was strongly reduced (CatCh-signal decrease of ~30%). As in CatCh alone a similar decrease (~20%) was observed but no displacement was observed in the imaging data (S3 Fig), we assume that the major part of the slight current decrease of tCXCR4/CatCh most likely corresponded to run-down effects in CatCh. Such initial current decrease can frequently be noticed in patch-clamp experiments under saturating light conditions.

Once internalized, the tCXCR4/CatCh endosomes migrate into the cell and serve as Ca^2+^ stores that can release Ca^2+^ into the cytosol upon blue light illumination. Assuming a mean radius of 500 nm for endosomes (Figs 2(B), 5(A) and 5(B)), the single channel conductance of CatCh of 140 fS [12], and a mean number [24] of 500 channel proteins per μm^2^ we can estimate the amount of Ca^2+^ ions released by each endosome. During the endocytosis process a substantial amount of Ca^2+^ can enter the cell and the vesicles are filled with extracellular medium. Once internalized, Ca^2+^ is quickly released from the endosome, which in turn is acidified [16,37]. The Ca^2+^ concentrations measured inside the endosomes differ strongly [38–40]. Assuming a Ca^2+^ concentration of 40 μM [10] the endosome contains around 12600 Ca^2+^ ions which could be released with a maximal rate of 7*10^6^ s^-1^ (see complete calculation in S1 Text). It has to be taken into account that, though CatCh provides increased permeability for Ca^2+^, other cations as sodium and protons have higher permeability and therefore might interfere with the Ca^2+^ signal. During endosome maturation the Ca^2+^ content increases again [39], thus allowing for an intensified light-induced Ca^2+^ signal by late endosomes. Though Albrecht et al. [39] postulate a minor role of endosomes in Ca^2+^ signal shaping, in comparison to mitochondria and ER, our theoretical considerations and experiments suggest endosomes as potential Ca^2+^ source for optophysiological experiments.

In accordance with our estimates, in intra-endosomal Ca^2+^ measurements (Fig 5A–5C) a non-linear decrease in rhod2 fluorescence was observed upon blue-light illumination in tCXCR4/CatCh endosomes, suggesting a release of Ca^2+^ ions into the cytosol, while only a slight linear decrease was observed in endosomes with CXCR4 alone. Indeed, measurements with rhod2-AM further supported the finding that the release of Ca^2+^ from the endosomes leads to an increase of cytosolic Ca^2+^ (Fig 5(D)) as the effect of tCXCR4/CatCh was quite pronounced though it was measured in absence of extracellular Ca^2+^. These measurements were performed in the presence of the CXCR4 antagonist AMD3100 in order to reduce the native signaling activity of CXCR4. Stimulation of CXCR4 will activate heterotrimeric G_i_ protein pathways and thus might affect the phosphorylation status of proteins or even influence the activity of Ca^2+^ channels [41–43]. Therefore, one is obligated to perform accurate control measurements deciphering the signals potentially induced by CXCR4 from those induced by Ca^2+^ elevation via the optochemokine tandem. This is especially important as due to the expression of the optochemokine tandem the number of CXCR4 copies in the cell increases. Further work is needed to optimize the tandem function for its use in a certain physiological process, e.g. by substituting CXCR4 by other class A GPCRs or by introducing mutations that affect the GPCRs function [44]. Furthermore, cell specific calcium release would require fine tuning of the tandem-signal activation process for the cell type used. Nevertheless, in our study the increment of cytosolic Ca^2+^ after illumination of the optochemokine tandem was clearly dependent on blue light (activation of ChR2) while with red light (no ChR2 activity) no difference was observed when compared with cells expressing CXCR4 only (S10 Fig).

## Conclusions

Summarizing our findings, the novel optophysiological tool combines two different features in one functional protein. First, the internalization pattern of distinct GPCRs can be followed up by the activity of CatCh in patch-clamp experiments. Thus, combining unknown or less investigated GPCRs with ChR2 in the tandem cassette would provide new insights into their internalization characteristics. Second, the intracellular Ca^2+^ concentration can be manipulate by light-triggered Ca^2+^ release from endosomes. In this respect it is important to emphasize that the tCXCR4/CatCh approach could be extended to combine different GPCRs with other (e.g. red-light sensitive) channelrhodopsins or even with various light-gated ion pumps suited for the modulation of intracellular pH, as recently done in synapses [45]. Time-resolved modulation of the Ca^2+^ concentration by light could be used to induce Ca^2+^-dependent processes like gating of Ca^2+^-activated Ca^2+^ channels, membrane fusion, secretion, induction of gene expression, and cell proliferation [8,46] or even cell death [47,48]. Indeed, in tCXCR4/CatCh expressing NG108-15 cells, as shown in S12 Fig, cell death was induced under physiological conditions by blue light reflecting its potential usefulness in the treatment of solid tumors. This topic will be subject of ensuing study to unravel a potential role of this optophysiological tool as a strategy for the induction of cell death of cancer cells upon light irradiation.

## Supporting Information

S1 Fig Cell cycle analysis of NG108-15 cells determined by flow cytometry. Illustration of the characteristic distribution of cells across the cell cycle suggesting absence of any chromosomal aberrant subpopulations. Experiments were performed with a BD FACSCanto™ II (BD Biosciences). The cells were detached by trypsination and centrifuged at 20°C and 400 g for 5 min. 5∙10^5^ cells were resuspended in DNA-buffer (100 mM Tris pH7.4, 154 mM NaCl, 1 mM CaCl_2_, 0.5 mM MgCl_2_, 0.2% BSA, 0.1% NP40, RNase A 100 U/ml and propidium iodide 10 μg/ml) and incubated for one hour at 4°C in the dark. Excitation of propidium iodide was performed with the 488 nm laser line. Data analysis and visualisation was performed with Flowing Software (version 2.5.1 Turku Centre for Biotechnology).
(PDF)

S2 Fig Electrorotation data of NG108-15 cells before (red) and after (black) treatment with SDF1α. C_m_ was calculated to be 2.46 ± 0.19 μF/cm^2^ (untreated cells, n = 366) and 1.89 ± 0.08 μF/cm^2^ (treated, n = 345), reflecting a significant decrease of the specific membrane capacitance upon addition of SDF1 (two tailed student t-test, alpha<0.5). Due to methodical reasons the absolute C_m_ values are slightly higher than in patch-clamp experiments (Fig 2), whereas the relative decrease is similar in both methods.
(PDF)

S3 Fig Confocal laser scanning micrographs of NG108-15 cells expressing ChR2::eYFP. Cells were imaged in presence of the CXCR4 inhibitor AMD3100 (left) or the activator SDF1α (right). Note that upon addition of SDF1 no substantial internalization of ChR2 could be observed. Scale bar represents 10 μm.
(PDF)

S4 Fig HEK293 CXCR4::eYFP cell line. Cells were incubated for 24 hours in media supplemented with either 10 μM AMD3100 (left) or 50 nM SDF1α (right). Strong internalization was observed in presence of the agonist but not the antagonist. Scale bar represents 20 μm.
(PDF)

S5 Fig HEK293 FlipIn-T-Rex tCXCR4/CatCh cell line. **a.** Confocal laser scanning micrographs showing co localisation of tCXCR4/CatCh (cyan) and antiCXCR4 antibody (red) after treatment with 50 nM SDF1α. White bar represents 5 μm. **b.** Patch-clamp experiments. Voltage step protocol in whole cell mode, showing full functionality of the CatCh-Protein in tCXCR4/CatCh.
(PDF)

S6 Fig Western blot analysis with anti-CXCR4 antibody. Membrane fragments were isolated from NG108-15 cells expressing either CXCR4::YFP, tCXCR4/CatCh, or no heterologous protein (control) as indicated.
(PDF)

S7 Fig CLSM analysis of endocytosis in HEK293 cells expressing tCXCR4/CatCh. **a.** Coexpression of tCXCR4/CatCh (cyan) and the endosome marker Rab5-RFP. **b**. Cells were exposed to Alexa647-transferrin (red) which is internalised into endosomes via clathrin-mediated endocytosis. Note, that both proteins colocalize with tCXCR4/CatCh indicating the endosome-nature of the observed intracellular vesicles. Scale bars represent 10 μm.
(PDF)

S8 Fig Time course of the closing kinetics of CatCh (black) and tCXCR4/CatCh (red). Patch-clamp experiments were performed at -100 mV membrane potential after blue light (473 nm; 200 ms) illumination in cell attached mode in presence of 50 nm SDF1α at 34–36°C. Mean τ_off_ and standard error of up to 10 cells (CatCh) and up to 14 cells (tCXCR4/CatCh) are given. There was no significant difference observable in the behavior of CatCh in the tandem construct compared to the protein alone. Note, that time constant differs from the published [12] value of 16 ms due to higher temperatures.
(PDF)

S9 Fig **Dependency of the tCXCR4/CatCh internalization on concentration (a) and splicing variant (b) of the chemokine ligand**. Data were obtained in cell attached Patch-clamp measurements of NG108-15 cells expressing tCXCR4/CatCh protein. **a.** When supplying 100 nM SDF1α instead of 50 nM the internalization efficiency did not significantly increase. Therefore, in our experiments we used 50 nM SDF1α. Mean values and standard error of 5 cells (50 nM SDF1α) and 3 cells (100 nM SDF1α) are given. **b.** No significant difference was observed between SDF1α (black) and SDF1β (red). Mean values and standard error of 5 cells (SDF1α) and 3 cells (SDF1β) are given.
(PDF)

S10 Fig Control experiment showing the time course of rhod2 fluorescence in endosomes exhibiting tCXCR4/CatCh(D156C) (7 cells, mean s.e.m) or CXCR4 (10 cells) upon red light illumination. The conditions were similar to the experiment depicted in Fig 5C but repetitive red light illumination (639 nm 5mW 4%) was used instead of blue light to avoid activation of CatCh.
(PDF)

S11 Fig Calcium-imaging of NG108-15 cells loaded with the calcium-dye rhod2-AM. Cells were transfected with tCXCR4/CatCh(D156C) (upper row, cyan) or CXCR4::eYFP (lower row, cyan) and pre-incubated with 50 nM SDF1α 24 h after expression before they were additionally loaded with the dye. Images were acquired by cLSM in absence of extracellular Ca^2+^. Each figure shows the fluorescence intensity of rhod2-AM (red) in ROIs containing either endosomes (white) or nucleus (magenta). Scale bars represent 10 μm. The graph below each photograph represents the time course of normalised fluorescence intensity ΔF/F_0_ within the respective ROIs as indicated. Averaged data of 5 cells are given in main document Fig 5D. See also S1 Movie.
(PDF)

S12 Fig Light-induced cell death in NG108-15 cells expressing tCXCR4/CatCh or CXCR4. **a.** Obvious changes in cell morphology were taken as criterion to distinguish dead and alive fluorescent cells. While healthy viable cell did not lose their membrane integrity, dead cells were recognized by membrane blebbing, cell shrinkage, and lose of cellular material (red arrows). **b.** Cells were illuminated with a light intensity of about 5 mW/ mm^2^ for 20 min followed by a subsequent dark exposure time of 40 min and the ratio of dead cells was followed over the time **c.** After 60 min the tCXCR4/CatCh cells showed a significant increase (two tailed student t-test, a<0.05) in the number of dead cells upon illumination as compared with CXCR4-expressing cells.
(PDF)

S1 Movie Intracellular, light-induced Ca^2+^-signaling. Ca^2+^ signals were mediated in NG108-15 cells by tCXCR4/CatCh (green) endosomes visualized by the calcium sensitive dye Rhod2-AM (red) and recorded with a confocal laser scanning microscope. In order to improve the signal to noise ratio of the rhod2 channel, the images were subjected to the Fiji mean filter (value = 1) and rhod2 intensity values are depicted on a logarithmic scale.
(MP4)

S1 Text Estimation of endosomal Ca^2+^ content and release rate. (PDF)
