# Supplementary figures and images for: Optochemokine Tandem for Light-Control of Intracellular Ca2+
Source: PLoS One. 2016 Oct 21;11(10):e0165344. doi: 10.1371/journal.pone.0165344 (PMC5074463; doi:10.1371/journal.pone.0165344)

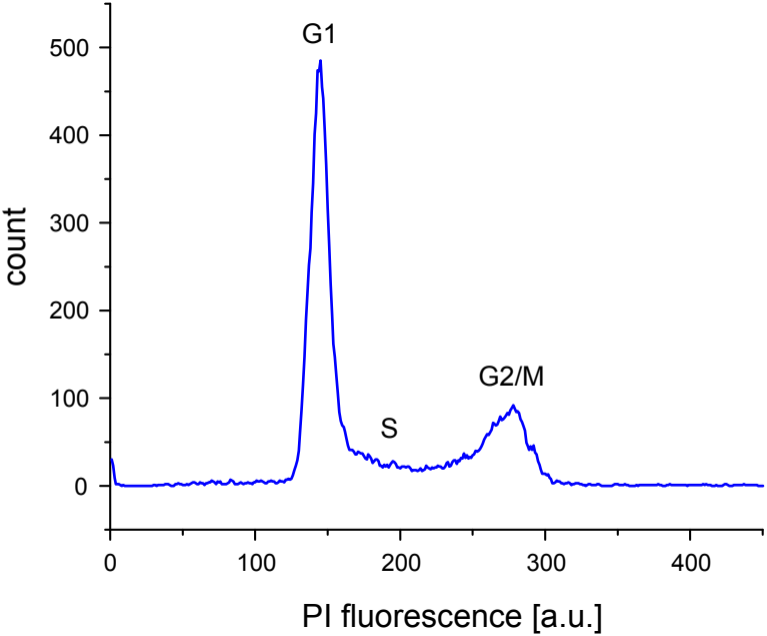

Supplement: S1 Fig — Illustration of the characteristic distribution of cells across the cell cycle suggesting absence of any chromosomal aberrant subpopulations. Experiments were performed with a BD FACSCanto™ II (BD Biosciences). The cells were detached by trypsination and centrifuged at 20°C and 400 g for 5 min. 5∙105 cells were resuspended in DNA-buffer (100 mM Tris pH7.4, 154 mM NaCl, 1 mM CaCl2, 0.5 mM MgCl2, 0.2% BSA, 0.1% NP40, RNase A 100 U/ml and propidium iodide 10 μg/ml) and incubated for one hour at 4°C in the dark. Excitation of propidium iodide was performed with the 488 nm laser line. Data analysis and visualisation was performed with Flowing Software (version 2.5.1 Turku Centre for Biotechnology). (PDF) [file pone.0165344.s001.pdf]

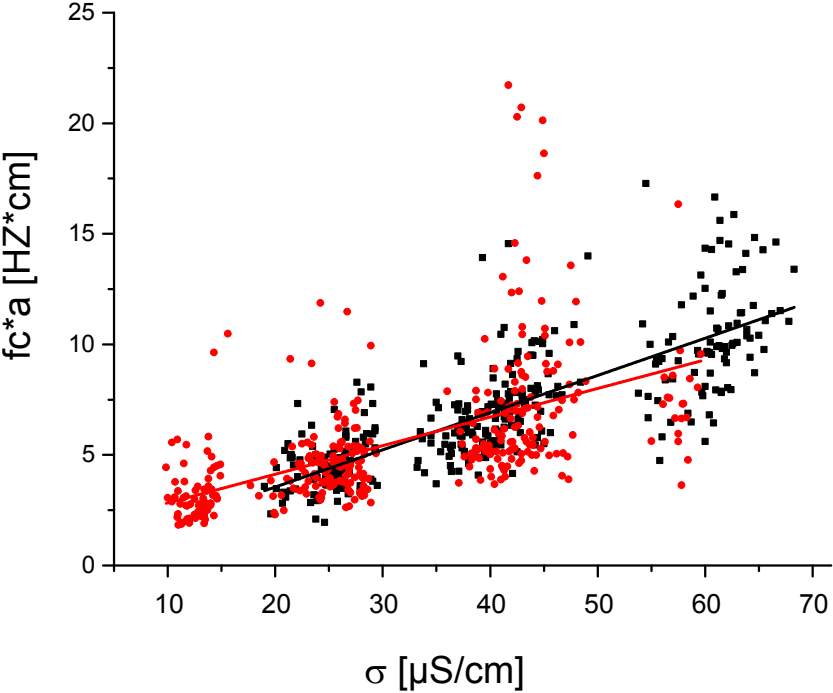

Supplement: S2 Fig — Cm was calculated to be 2.46 ± 0.19 μF/cm2 (untreated cells, n = 366) and 1.89 ± 0.08 μF/cm2 (treated, n = 345), reflecting a significant decrease of the specific membrane capacitance upon addition of SDF1 (two tailed student t-test, alpha<0.5). Due to methodical reasons the absolute Cm values are slightly higher than in patch-clamp experiments (Fig 2), whereas the relative decrease is similar in both methods. (PDF) [file pone.0165344.s002.pdf]

AMD3100  
10  $\mu$ M

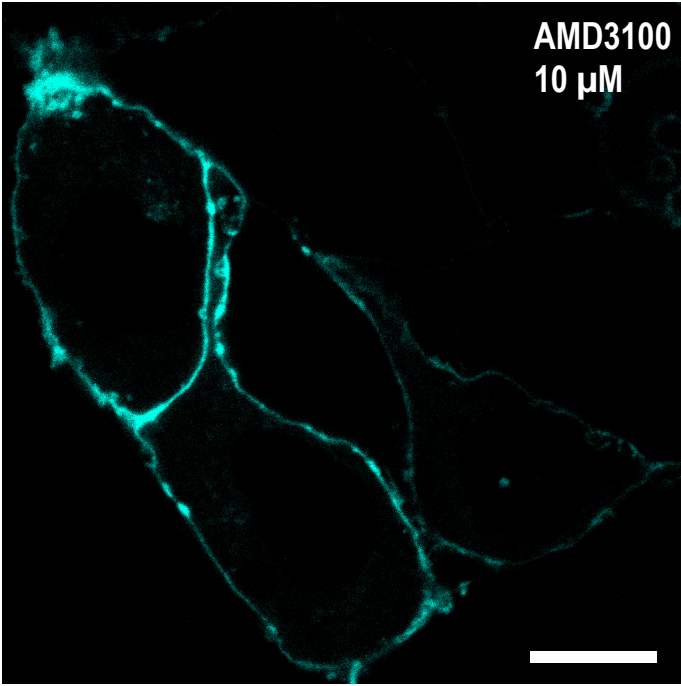

SDF1 $\alpha$   
50 nM

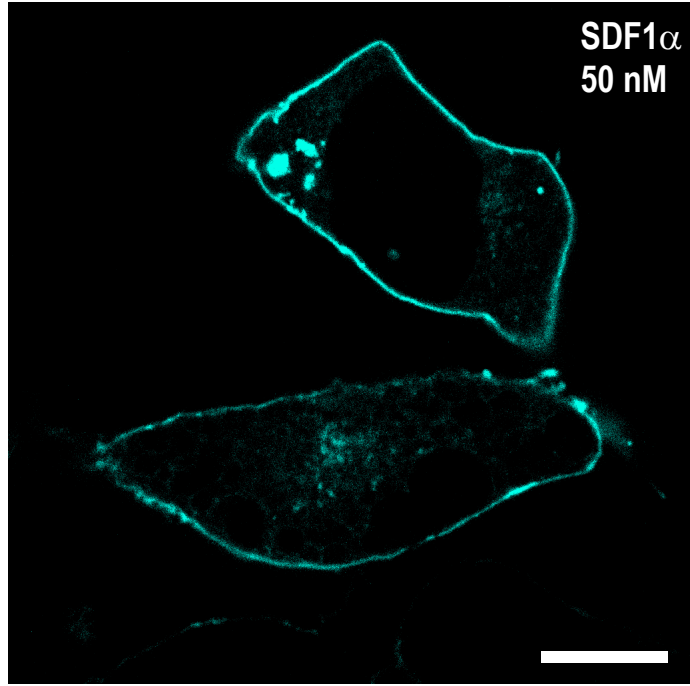

Supplement: S3 Fig — Cells were imaged in presence of the CXCR4 inhibitor AMD3100 (left) or the activator SDF1α (right). Note that upon addition of SDF1 no substantial internalization of ChR2 could be observed. Scale bar represents 10 μm. (PDF) [file pone.0165344.s003.pdf]

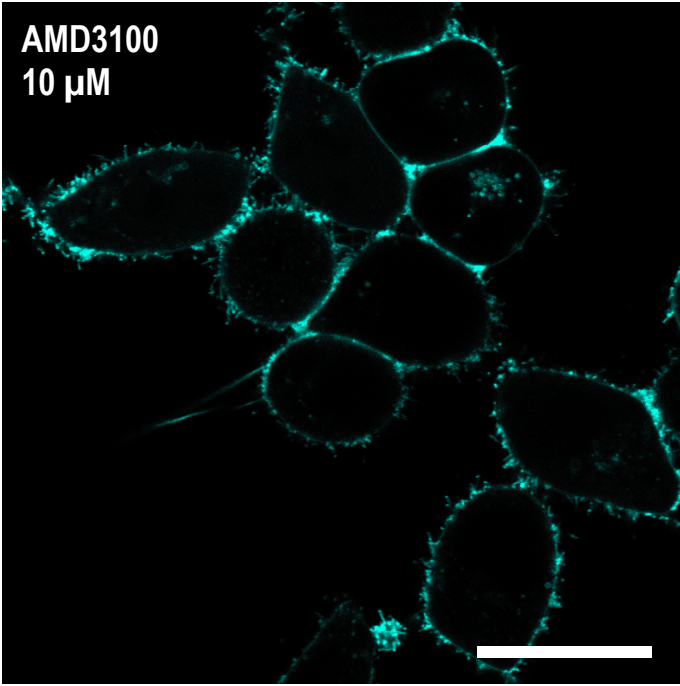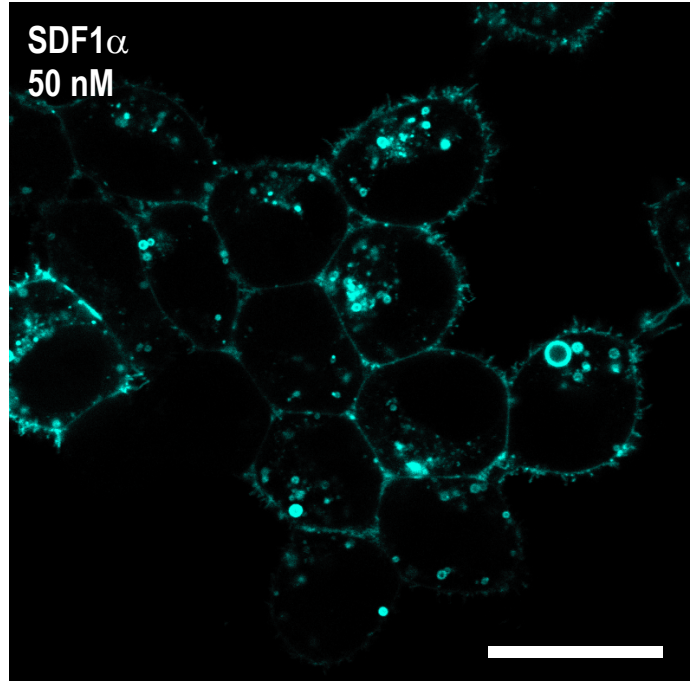

Supplement: S4 Fig — Cells were incubated for 24 hours in media supplemented with either 10 μM AMD3100 (left) or 50 nM SDF1α (right). Strong internalization was observed in presence of the agonist but not the antagonist. Scale bar represents 20 μm. (PDF) [file pone.0165344.s004.pdf]

a

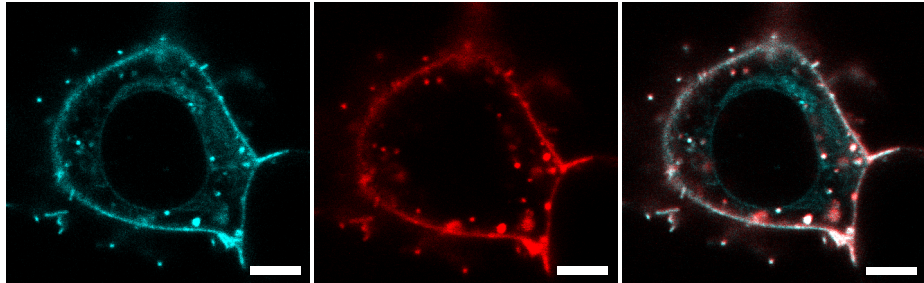

b

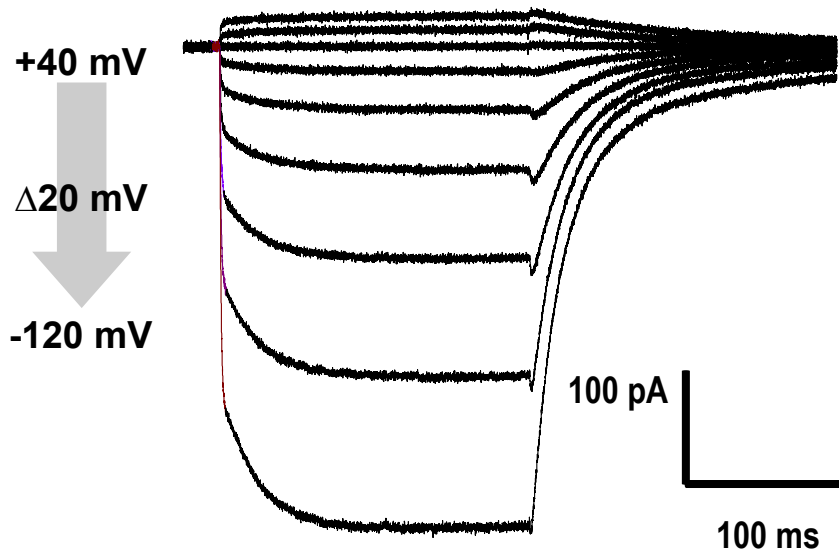

Supplement: S5 Fig — a. Confocal laser scanning micrographs showing co localisation of tCXCR4/CatCh (cyan) and antiCXCR4 antibody (red) after treatment with 50 nM SDF1α. White bar represents 5 μm. b. Patch-clamp experiments. Voltage step protocol in whole cell mode, showing full functionality of the CatCh-Protein in tCXCR4/CatCh. (PDF) [file pone.0165344.s005.pdf]

kDa

140  
115

80

65

50

40

30

25

CXCR4-YFP

tCXCR4/CatCh

Control

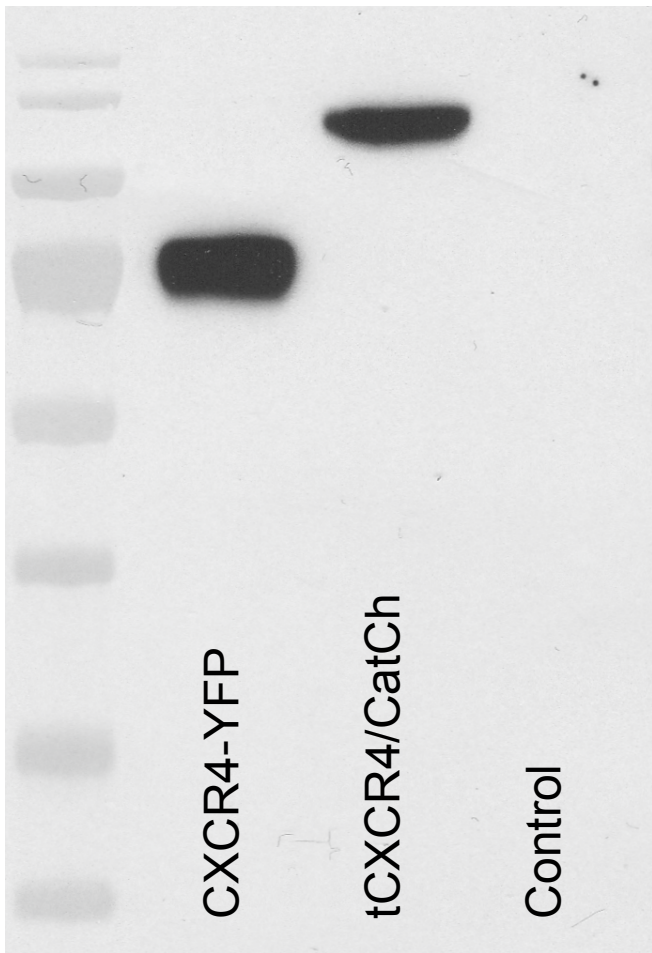

Supplement: S6 Fig — Membrane fragments were isolated from NG108-15 cells expressing either CXCR4::YFP, tCXCR4/CatCh, or no heterologous protein (control) as indicated. (PDF) [file pone.0165344.s006.pdf]

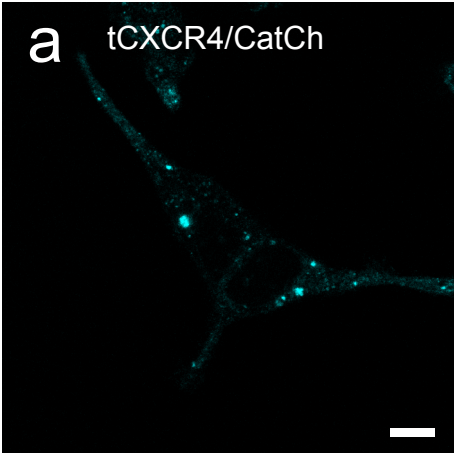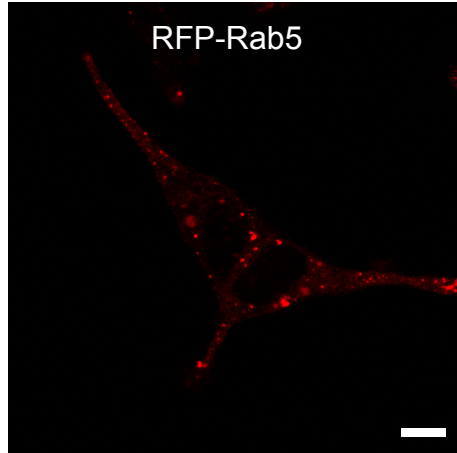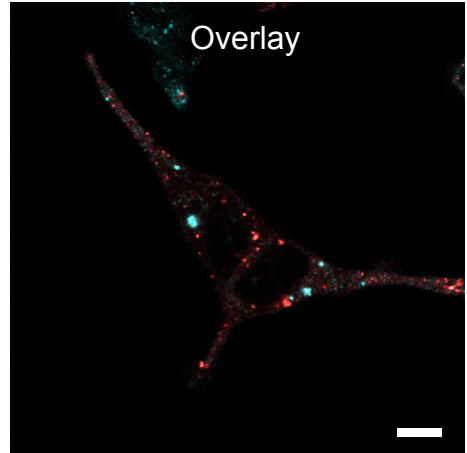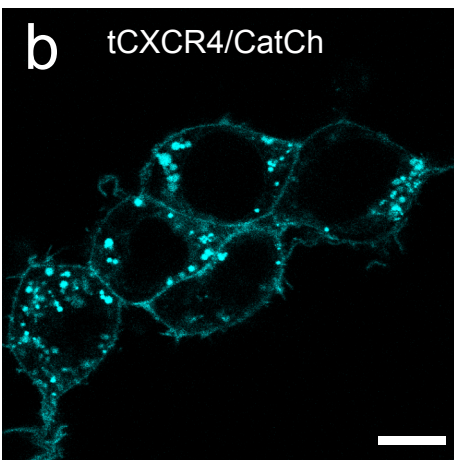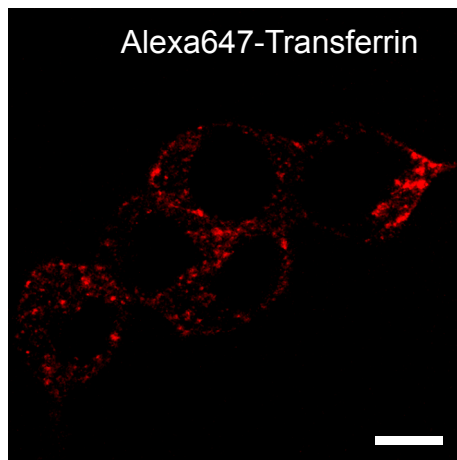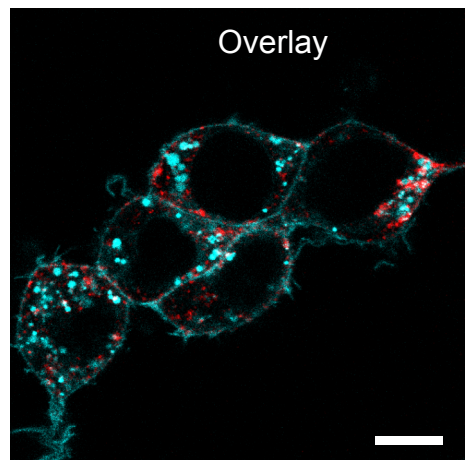

Supplement: S7 Fig — a. Coexpression of tCXCR4/CatCh (cyan) and the endosome marker Rab5-RFP. b. Cells were exposed to Alexa647-transferrin (red) which is internalised into endosomes via clathrin-mediated endocytosis. Note, that both proteins colocalize with tCXCR4/CatCh indicating the endosome-nature of the observed intracellular vesicles. Scale bars represent 10 μm. (PDF) [file pone.0165344.s007.pdf]

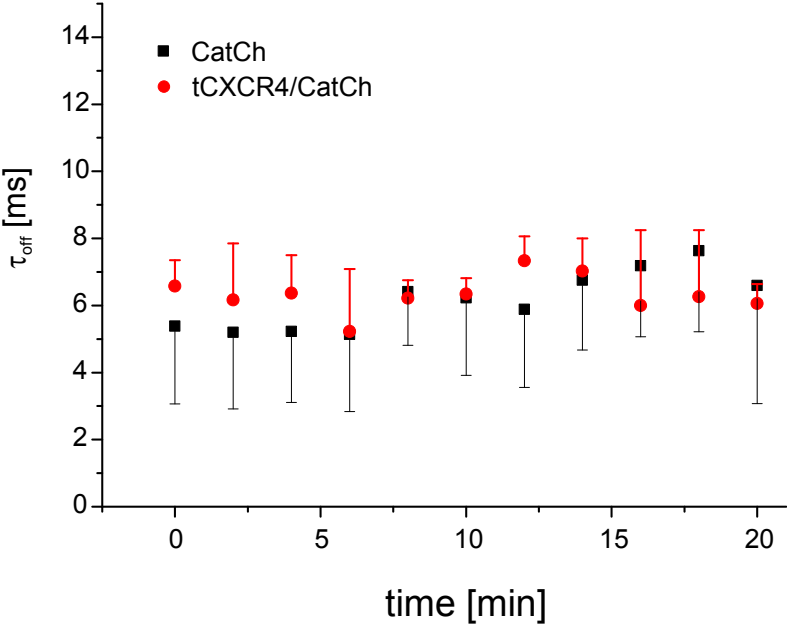

Supplement: S8 Fig — Patch-clamp experiments were performed at -100 mV membrane potential after blue light (473 nm; 200 ms) illumination in cell attached mode in presence of 50 nm SDF1α at 34–36°C. Mean τoff and standard error of up to 10 cells (CatCh) and up to 14 cells (tCXCR4/CatCh) are given. There was no significant difference observable in the behavior of CatCh in the tandem construct compared to the protein alone. Note, that time constant differs from the published [12] value of 16 ms due to higher temperatures. (PDF) [file pone.0165344.s008.pdf]

a

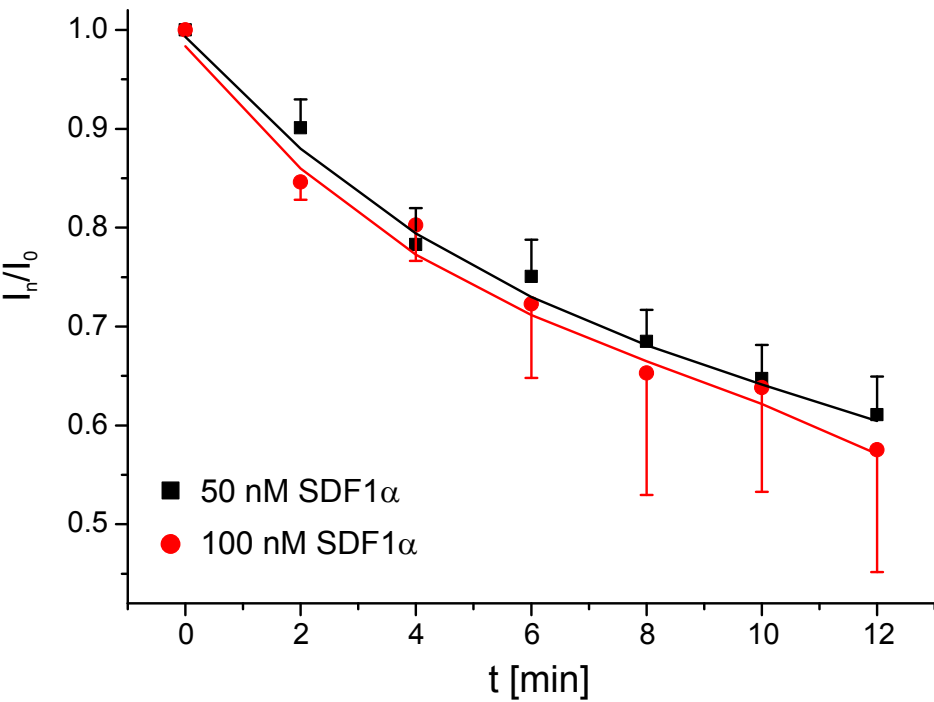

b

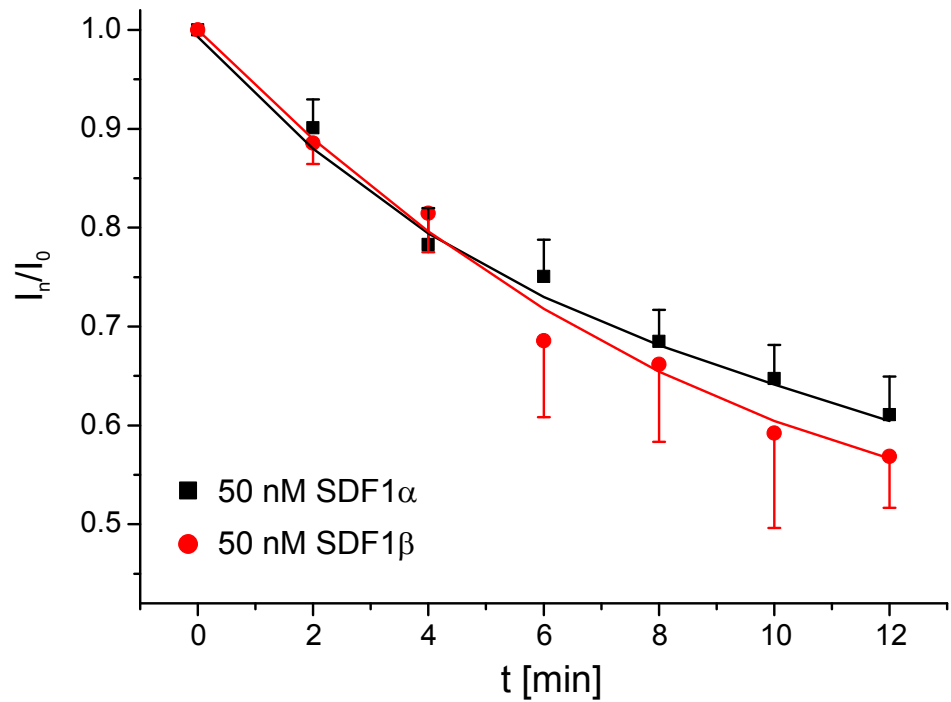

Supplement: S9 Fig — Dependency of the tCXCR4/CatCh internalization on concentration (a) and splicing variant (b) of the chemokine ligand. Data were obtained in cell attached Patch-clamp measurements of NG108-15 cells expressing tCXCR4/CatCh protein. a. When supplying 100 nM SDF1α instead of 50 nM the internalization efficiency did not significantly increase. Therefore, in our experiments we used 50 nM SDF1α. Mean values and standard error of 5 cells (50 nM SDF1α) and 3 cells (100 nM SDF1α) are given. b. No significant difference was observed between SDF1α (black) and SDF1β (red). Mean values and standard error of 5 cells (SDF1α) and 3 cells (SDF1β) are given. (PDF) [file pone.0165344.s009.pdf]

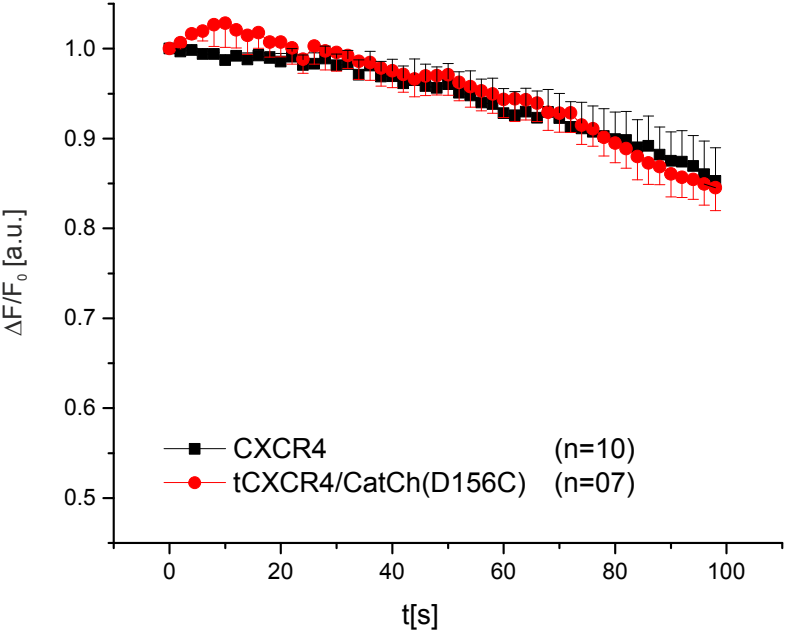

Supplement: S10 Fig — The conditions were similar to the experiment depicted in Fig 5C but repetitive red light illumination (639 nm 5mW 4%) was used instead of blue light to avoid activation of CatCh. (PDF) [file pone.0165344.s010.pdf]

# tCXCR4/CatCh(D156C)

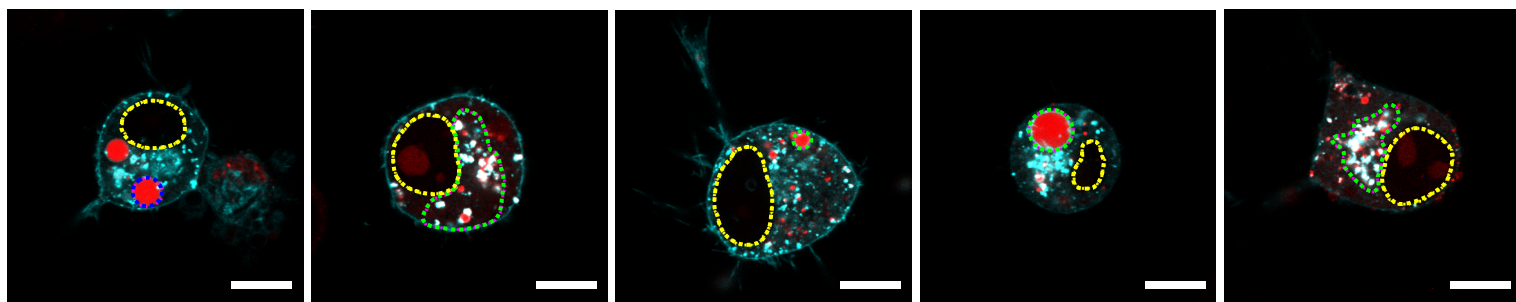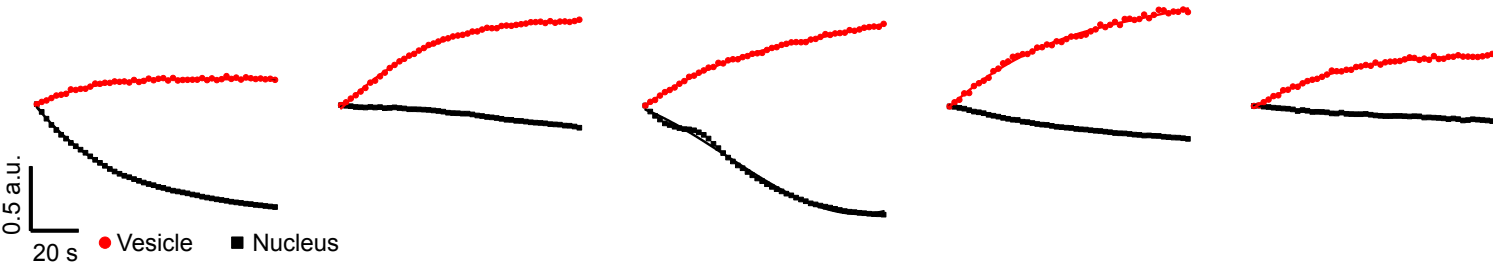

# CXCR4

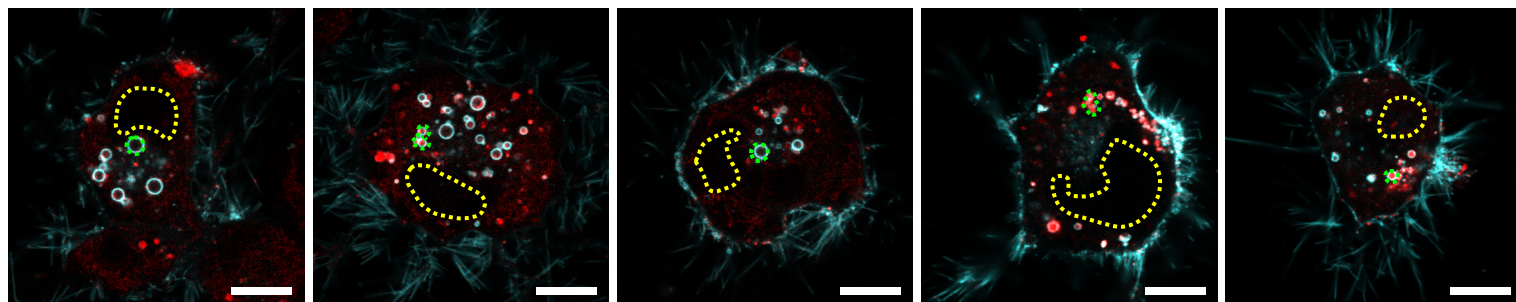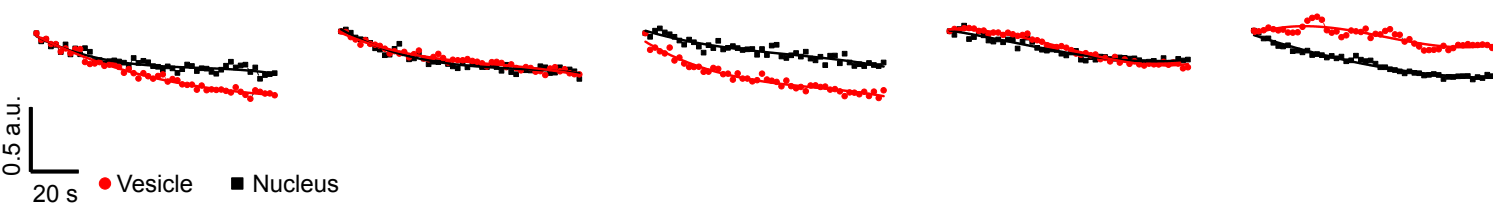

Supplement: S11 Fig — Cells were transfected with tCXCR4/CatCh(D156C) (upper row, cyan) or CXCR4::eYFP (lower row, cyan) and pre-incubated with 50 nM SDF1α 24 h after expression before they were additionally loaded with the dye. Images were acquired by cLSM in absence of extracellular Ca2+. Each figure shows the fluorescence intensity of rhod2-AM (red) in ROIs containing either endosomes (white) or nucleus (magenta). Scale bars represent 10 μm. The graph below each photograph represents the time course of normalised fluorescence intensity ΔF/F0 within the respective ROIs as indicated. Averaged data of 5 cells are given in main document Fig 5D. See also S1 Movie. (PDF) [file pone.0165344.s011.pdf]

**a**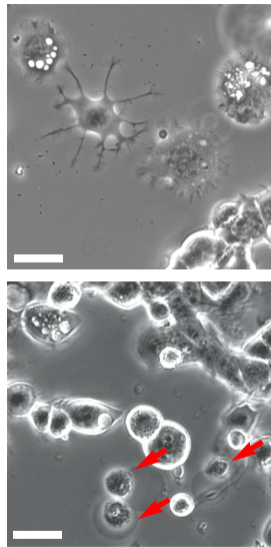**b**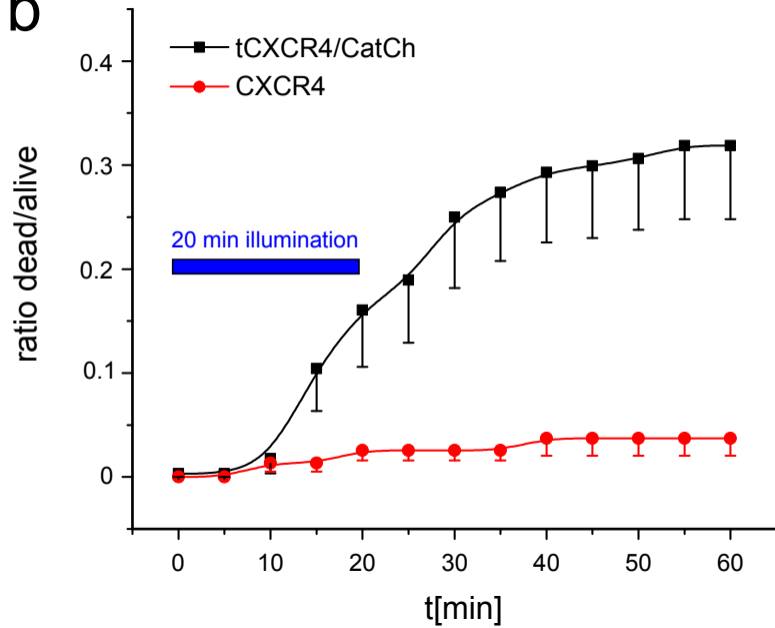**c**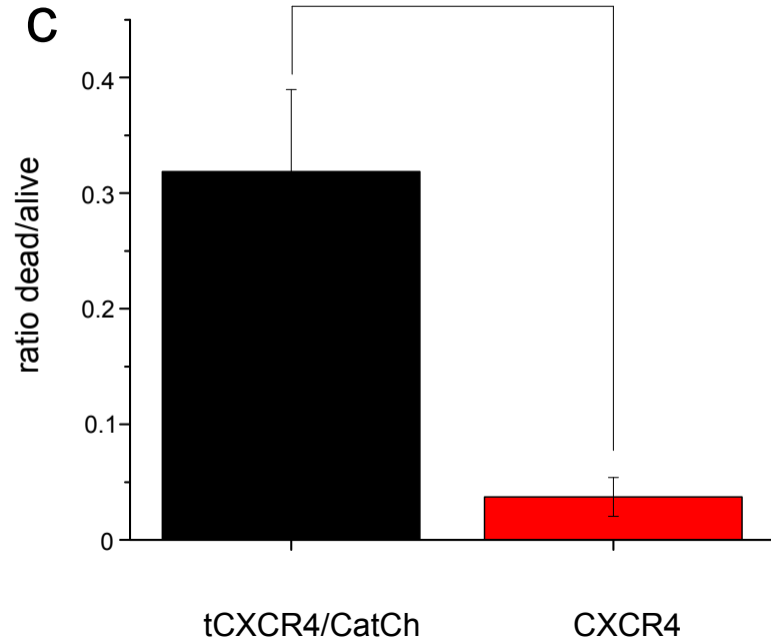

Supplement: S12 Fig — a. Obvious changes in cell morphology were taken as criterion to distinguish dead and alive fluorescent cells. While healthy viable cell did not lose their membrane integrity, dead cells were recognized by membrane blebbing, cell shrinkage, and lose of cellular material (red arrows). b. Cells were illuminated with a light intensity of about 5 mW/ mm2 for 20 min followed by a subsequent dark exposure time of 40 min and the ratio of dead cells was followed over the time c. After 60 min the tCXCR4/CatCh cells showed a significant increase (two tailed student t-test, a<0.05) in the number of dead cells upon illumination as compared with CXCR4-expressing cells. (PDF) [file pone.0165344.s012.pdf]
