# Supplementary material for: Optochemokine Tandem for Light-Control of Intracellular Ca2+
Source: PLoS One. 2016 Oct 21;11(10):e0165344. doi: 10.1371/journal.pone.0165344 (PMC5074463; doi:10.1371/journal.pone.0165344)
Supplement: S1 Text — (PDF) [file pone.0165344.s014.pdf]

S1 Text. Estimation of endosomal Ca^{2+} content and release rate

For the estimation of the Ca^{2+} amount that can be released from an endosome upon illumination the following parameters are assumed:

C_i	Cytosolic Ca^{2+} concentration	100 nM
C_e	Endosomal Ca^{2+} concentration [2]	40 µM
σ	Single channel conductance of CatCh [1]	140 fS
n	Estimated number of proteins [3]	500 / µm²
A	Endosome surface	3.1416 µm²
r	Endosome radius	0.5 µm
p	open probability	0.5
E	Membrane potential Endosome [4]	20 mV
z	valence of Ca^{2+}	2
e	elementary charge	$1.60218 * 10^{-19}$ C
N_A	Avogadro constant	$6.02214*10^{23}$ mol^{-1}

The total amount of Ca^{2+} ions present in one endosome can be estimated assuming a spherical shape:

$$n_{Ca2+} = \frac{4}{3}\pi r^3 * c_e * N_A = \frac{4}{3}\pi(5 * 10^{-7})^3 m^3 * 0.04 \frac{mol}{m^3} * 6.02214 * 10^{23}\ mol^{-1} = 12613$$

The rate at which ions leave the endosome through tCXCR4/CatCh into the cytosol right upon illumination of the cell can be estimated by the following formula:

$$J_{Ca2+} = E * n * A * \sigma * p * (ze)^{-1} = 0.02\ V * 500 * 3.1416 * 140 * 10^{-15} C(sV)^{-1} * 0.5 * (2 * 1.60218 * 10^{-19}\ \mathrm{C})^{-1} = 6.86 * 10^{6}\ s^{-1}$$

Thus, the amount of calcium released from the endosomes is mainly limited by the number of ions present rather than by the permeability of CatCh.

1. Kleinlogel S, Feldbauer K, Dempski RE, Fotis H, Wood PG, Bamann C, et al. Ultra light-sensitive and fast neuronal activation with the Ca2+-permeable channelrhodopsin CatCh. Nat Neurosci. 2011;14: 513–518. doi:10.1038/nn.2776
2. Lloyd-Evans E, Waller-Evans H, Peterneva K, Platt FM. Endolysosomal calcium regulation and disease. Biochem Soc Trans. 2010;38: 1458–1464. doi:10.1042/BST0381458
3. Zimmermann D, Zhou A, Kiesel M, Feldbauer K, Terpitz U, Haase W, et al. Effects on capacitance by overexpression of membrane proteins. Biochem Biophys Res Commun. 2008;369: 1022–1026. doi:doi:10.1016/j.bbrc.2008.02.153
4. Koivusalo M, Steinberg BE, Mason D, Grinstein S. In situ Measurement of the Electrical Potential Across the Lysosomal Membrane Using FRET. Traffic. Blackwell Publishing Ltd; 2011;12: 972–982. doi:10.1111/j.1600-0854.2011.01215.x
